# Multilocus Association Testing of Quantitative Traits Based on Partial Least-Squares Analysis

**DOI:** 10.1371/journal.pone.0016739

**Published:** 2011-02-03

**Authors:** Feng Zhang, Xiong Guo, Hong-Wen Deng

**Affiliations:** 1 Key Laboratory of Environment and Gene Related Diseases of Ministry Education, Faculty of Public Health, College of Medicine, Xi'an Jiaotong University, Xi'an, Shaanxi, People's Republic of China; 2 Center of System Biomedical Sciences, Shanghai University of Science and Technology, Shanghai, People's Republic of China; 3 Departments of Orthopedic Surgery and Basic Medical Science, School of Medicine, University of Missouri-Kansas City, Kansas City, Missouri, United States of America; Innsbruck Medical University, Austria

## Abstract

Because of combining the genetic information of multiple loci, multilocus association studies (MLAS) are expected to be more powerful than single locus association studies (SLAS) in disease genes mapping. However, some researchers found that MLAS had similar or reduced power relative to SLAS, which was partly attributed to the increased degrees of freedom (dfs) in MLAS. Based on partial least-squares (PLS) analysis, we develop a MLAS approach, while avoiding large dfs in MLAS. In this approach, genotypes are first decomposed into the PLS components that not only capture majority of the genetic information of multiple loci, but also are relevant for target traits. The extracted PLS components are then regressed on target traits to detect association under multilinear regression. Simulation study based on real data from the HapMap project were used to assess the performance of our PLS-based MLAS as well as other popular multilinear regression-based MLAS approaches under various scenarios, considering genetic effects and linkage disequilibrium structure of candidate genetic regions. Using PLS-based MLAS approach, we conducted a genome-wide MLAS of lean body mass, and compared it with our previous genome-wide SLAS of lean body mass. Simulations and real data analyses results support the improved power of our PLS-based MLAS in disease genes mapping relative to other three MLAS approaches investigated in this study. We aim to provide an effective and powerful MLAS approach, which may help to overcome the limitations of SLAS in disease genes mapping.

## Introduction

Association studies are widely used to identify genetic variants underlying complex human diseases, such as osteoporosis [1], [2], obesity [3] and diabetes [4]. Association studies can be generally classified into two classes: single locus association studies (SLAS) and multiple loci association studies (MLAS) [5]. SLAS detect associations between each individual locus and target traits. Because of being simple to implement, SLAS are popular in current association mapping of disease genes. However, there are several limitations for SLAS. First, the performance of SLAS largely depends on the linkage disequilibrium (LD) between testing loci and potential causal loci. SLAS may have low power if the LD between testing loci and potential causal loci is weak. Second, it is well known that the risks of complex human diseases are usually determined by the main and interactive effects of multiple genetic and environmental factors [6]. Because SLAS conduct association tests at each individual locus, it is difficult to detect genetic interactive effects using SLAS. Third, association studies usually request a multiple testing adjustment procedure to ensure overall appropriate type I error rates, such as Bonferroni correction [7], [8] and false discovery rates [9], [10], [11]. These multiple testing adjustment procedures are sometimes too strict, and may miss real disease-gene associations in large scale SLAS.

The limitations of SLAS promote the development of MLAS approaches. Because MLAS can simultaneously consider the genetic information of multiple loci, it is expected that MLAS were more powerful than SLAS in disease genes mapping. Multilinear regression is one of the major multivariate analyses approaches, and has been applied to MLAS [12], [13]. In multilinear regression, target trait values can be modeled as a function of independent variable vector corresponding to the genotypes of multiple loci in candidate genetic regions. Because of large degrees of freedom (dfs) in statistical tests, it is difficult to directly apply multilinear regression to large genetic regions for MLAS. Previous studies found that multilinear regression had similar or reduced power relative to SLAS in disease gene mapping [14], [15], [16]. The increased power gained from combining the genetic information of multiple loci may be compromised by increasing dfs in multilinear regression. Additionally, the genotypes of multiple densely spaced loci are usually correlated due to LD, which may induce collinearity of genotype vectors, and decrease the power of multilinear regression for MLAS [12].

Several methods have been proposed to deal with large dfs in multilinear regression. The first one is tagSNPs-based multilinear regression [14], [15]. A set of tagSNPs capturing majority of the genetic information of candidate genetic regions, and having no or weak collinearity among each other, can be selected and included into multilinear regression for MLAS. Although selecting tagSNPs can decrease dfs in multilinear regression, it will result in the lost of genetic information and therefore decrease the power of MLAS, especially in the genetic regions with weak LD. Additionally, the power of tagSNPs-based association studies is affected by the performance of tagSNPs selection methods [17], [18]. The second method applies dimension reduction techniques, such as principle component analysis (PCA) [12], [19] and Fourier transformation [20], to genotype data and produces a set of orthogonal predictors capturing majority of the genetic information of candidate genetic regions. One can then detect associations between the extracted orthogonal predictors and target traits under multilinear regression [12], [20]. Besides the multilinear regression-based MLAS approaches mentioned above, other MLAS approaches are also available, such as genetic similarity-based MLAS [21], [22] and Bayesian-based MLAS [23].

Recently, Taylor and Tibshirani proposed the tail strength measure (TSM) for assessing the overall significance levels of multiple hypotheses tests in microarray studies [24]. Using simulated and real microarray datasets, Taylor and Tibshirani illustrated the performance of TSM, and suggested that TSM could be used to assess overall significance levels in microarray and other genetic studies with a number of hypotheses tests [24]. TSM may be able to evaluate overall association strength of multiple loci in association studies. However, the performance of TSM for MLAS remains unclear.

In this paper, we present a MLAS approach based on partial least-squares (PLS) analysis, while avoiding large dfs. As an extension of multiple linear regression, PLS generalizes and combines the features of PCA and multilinear regression [25], [26]. Through maximizing the covariance of denpendent and indenpendent variables, PLS searches for the components capturing majority of the information contained in indenpendent variables as well as in the relations between denpendent and indenpendent variables. In Materials and Methods section, we first formulate our PLS-based MLAS. Using simulated data based on real data from the HapMap project, we show that PLS-based MLAS are simple to implement, and generally provides improved power in diseases genes mapping relative to tagSNPs-based MLAS, PCA-based MLAS and TSM-based MLAS. Finally, a real data is used to assess the performance of PLS-based MLAS for genome-wide MLAS.

## Results

### Simulations

**Figure 1:**
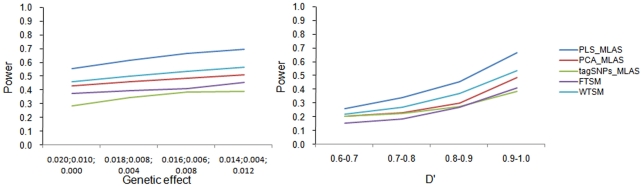
Power comparing results of PLS-based MLAS (PLS_MLAS), PCA-based MLAS (PCA_MLAS), tagSNPs-based MLAS (tagSNPs_MLAS), TSM-based MLAS using F test (FTSM) and TSM-based MLAS using Wald test (WTSM) under the epistatic model.

The type I error rates of the four MLAS approaches under various scenarios investigated in this study are normal and not shown to simplify our presentation. The power comparison results of the four MLAS approaches under the epistatic model are presented in Figure 1. As shown by the data, PLS-based MLAS attained the highest power, followed by WTSM and PCA-based MLAS across various parameter settings. TagSNPs-based MLAS and FTSM appeared to perform less well than other MLAS approaches in this study.

**Figure 2:**
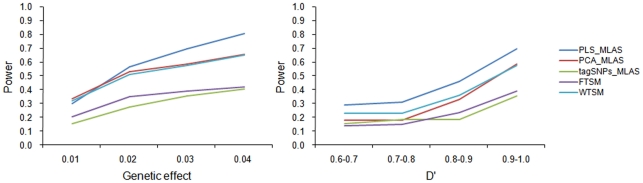
Power comparing results of PLS-based MLAS (PLS_MLAS), PCA-based MLAS (PCA_MLAS), tagSNPs-based MLAS (tagSNPs_MLAS), TSM-based MLAS using F test (FTSM) and TSM-based MLAS using Wald test (WTSM) under the additive model.

Figure 2 presents the power comparison results of the four MLAS approaches under the additive model. In the simulation study of genetic effect, PLS-based MLAS obtained higher power than other MLAS approaches under various genetic effects except for 0.01. WTSM and PCA-based MLAS showed similar power, and outperformed tagSNPs-based MLAS and FTSM. In the simulation study of D', PLS-based MLAS appeared to significantly perform better than other MLAS approaches investigated in this study.

### Genome-wide MLAS of Lean Body Mass

**Figure 3:**
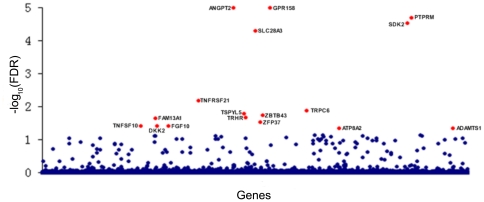
Plot of genome-wide MLAS results of lean body mass implemented by PLS-based MLAS. Significant genes are highlighted in red.

Figure 3 summarizes the genome-wide MLAS results of lean body mass implemented by PLS-based MLAS. Beside TRHR detected in previous genome-wide SLAS of lean body mass [27], we identified 16 novel genes with significant association signals for lean body mass. To evaluate the efficiency and robust of our PLS-based MLAS, we further compared the MLAS and SLAS results of the 17 genes detected in this study, as shown by Table 1.

**Table 1:** Comparison of MLAS and SLAS results of the 17 genes detected by PLS-based MLAS of lean body mass.

Genes	PLS-based MLAS		SLAS	
	P values	FDR	P values^a^	FDR
ADAMTS1	1.00×10^−5^	0.045	3.31×10^−5^	0.514
ANGPT2	2.00×10^−5^	1.00×10^−5^	4.76×10^−4^	0.906
ATP8A2	1.30×10^−4^	0.045	0.016	0.919
DKK2	3.00×10^−5^	0.037	5.66×10^−3^	0.910
FAM13A1	8.10×10^−4^	0.022	5.64×10^−4^	0.906
FGF10	1.00×10^−5^	0.037	1.16×10^−4^	0.753
GPR158	4.00×10^−5^	1.00×10^−5^	1.56×10^−4^	0.798
PTPRM	3.00×10^−5^	2.00×10^−5^	6.40×10^−3^	0.910
SDK2	7.00×10^−5^	3.00×10^−5^	0.029	0.935
SLC28A3	3.60×10^−4^	5.00×10^−5^	1.36×10^−3^	0.909
TNFRSF21	0.020	6.68×10^−3^	1.74×10^−4^	0.811
TNFSF10	3.00×10^−5^	0.039	0.020	0.919
TRHR	2.20×10^−3^	0.021	7.55×10^−8^	0.029
TRPC6	1.24×10^−3^	0.013	2.97×10^−3^	0.910
TSPYL5	1.90×10^−4^	0.016	1.62×10^−3^	0.910
ZBTB43	1.00×10^−5^	0.018	3.53×10^−3^	0.910
ZFP37	3.00×10^−5^	0.028	0.011	0.910

a denote the smallest P value of each gene obtained from our previous genome-wide SLAS of lean body mass.

## Discussion

Large dfs is one of the major issues with MLAS. To deal with this problem, we propose a PLS-based MLAS approach, while avoiding large dfs. Simulation study based on real data from the HapMap project suggests that our PLS-based MLAS generally outperformed other three popular MLAS approaches under various scenarios investigated in this study. PLS is suitable to handle the data with many independent variables as well as multicollinearity among the variables [28], [29], which are common in genotype data due to LD. It has been suggested that PLS might provide more genetic information than PCA do, when interactive effects [30] or multicollinearity [29] exist. In contrast, because PCA only consider the characteristics of indenpendent variables, the PCA components capturing major genetic information of candidate genetic regions are not necessarily relevant for target traits. Therefore, it is not surprising that PLS-based MLAS are more powerful than PCA–based MLAS in this study.

PLS-based MLAS can easily be applied to genome-wide association studies (GWAS). To investigate the performance of PLS-based MLAS for GWAS, we conducted a genome-wide MLAS of lean body mass using a real sample consisting of 973 unrelated USA whites. To the best of our knowledge, this is the first multilocus GWAS of lean body mass. Besides TRHR detected in previous genome-wide SLAS of lean body mass [27], PLS-based MLAS identified 16 novel genes that may be missed by previous study. Although the 16 genes did not achieve genome-wide significance level (1.32×10^−7^) in previous genome-wide SLAS of lean body mass, most of them still attained small p values (Table 1). Biological studies of these genes may provide some evidences for their roles in the genetic regulation of lean body mass. For instance, it has been found that serum TNFSF10 (also named TRAIL) concentration was significantly correlated with lean body mass [31]. TNFSF10 might play an important role in skeletal myoblast differentiation [32]. Rat experiments observed that ADAMTS1 was highly expressed in skeletal muscle [33], and muscular development appeared to rely on ADAMTS1 [34]. Replication studies are needed to validate the associations between the 17 genes and lean body mass detected in this study.

To illustrate the performance of our approach, we developed PLS-based MLAS for quantitative traits in this study. However, PLS-based MLAS can easily be extended to qualitative traits under logistic regression model. Covariates can also be incorporated into PLS-based MLAS due to the flexibility of regression analyses. Additionally, because permutations are used to evaluate the significance level of testing statistic, our PLS-based MLAS do not depend on specific statistical assumption, for instance the normality assumption of target traits. The computational cost of PLS-based MLAS is also acceptable for real studies. For instance, our genome-wide MLAS of lean body mass using PLS-based MLAS needed about 21 days (running on Dell computer cluster with four Intel Xeon 1.6 GHz processors and 4G memory).

An issue with PLS-based MLAS is how many PLS compoments we should include into analyses. Some methods developed for PCA can be used here. For instance, we can select top m of the ordered PLS components that explain certain proportions of total genotypic variance (for example, selecting top m PLS components explaining 80% of total genotypic variance) [12]. It should be noted that using too many components in PLS-based MLAS and PCA-based MLAS may also decrease the power of MLAS due to increasing dfs.

Taylor and Tibshirani originally proposed TSM to assess the overall significance levels of multiple hypotheses tests in microarray studies [24]. Here, we applied TSM to MLAS, and implemented a permutation procedure to estimate the empirical p value of TSM statistic. Although TSM-based MLAS performed less well than PLS-based MLAS, TSM is easy to calculate and may provide a simple alternative for MLAS. Additionally, we found that WTSM significantly outperformed FTSM in our simulation study, which suggest the impact of statistical tests used for calculating TSM on the performance of TSM-based MLAS. Based on our simulation study results, we suggest that it was better to use powerful statistical tests in TSM-based MLAS, such as Wald test and likelihood ratio test.

In summary, we present a simple and flexible MLAS approach with small dfs. Simulation study and real GWAS data analyses results support the improved performance of our PLS-based MLAS in disease genes mapping relative to other popular MLAS approaches investigated in this study. We aim to provide an effective and powerful MLAS approach, which may help to overcome the limitations of SLAS in disease genes mapping.

## Materials and Methods

### Ethics Statement

All studies were approved by the Institutional Review Boards of University of Missouri-Kansas City. Informed-consent documents were written by all study participants.

### Extended Tukey's 1-df interaction model

Consider a sample of n unrelated subjects with k genotyped SNPs. Let Y_i_ denote the quantitative trait value for subject i (i = 1,…,n), and X_ij_ denote the genotype of subject i at SNP j (j = 1,…,k). In this study, we coded X_ij_ to be 0, 1 or 2, representing the copy number of minor allele for subject i at SNP j. Other genotype coding scenarios can also be used, such as X_ij_  = 0 or 1 for genetic dominant or recessive models, if desired. The extended Tukey's 1-df interaction model proposed by Chatterjee N et al. [13] was implemented here to model the relationship between individual trait value Y_i_ and genotype X_ij_. In this model, the SNPs with large marginal genetic effects are assumed to have large interactive genetic effects. Total interactive effects of all SNPs are measured by weighted sum of the product of marginal effects of each pair of SNPs through an interaction parameter γ in the extended Tukey's 1-df interaction model, defined by
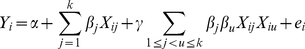
(1)where α is intercept. β_j_ denotes the regression coefficient for SNP j. γ measures total interactive effects of all SNPs within candidate genetic regions. e_i_ denotes the residual environmental effect for subject i, and is assumed to follow a normal distributation with mean 0 and variance 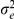. A global test of associations between candidate genetic regions and target traits equates testing the null hypothesis H_0:_ β = 0 (β = β_1_,…, β_k_) under multilinear regression.

Although the interactive effects of multiple SNPs are modeled as an interaction parameter γ in the extended Tukey's 1-df interaction model [13], it is still difficult to apply this model to large genetic regions with many SNPs. The parameters needed for large genetic regions in the extended Tukey's 1-df interaction model will become too many to implement.

### PLS-based MLAS

Instead of directly using genotypes as regressor, we propose to regress the PLS components derived from genotypes on target traits under multilinear regression model. A standard iterative process implemented by the pls package of R was used to derive PLS components from genotypes in this study [35], [36]. Briefly, let Y_j_ denote the residual phenotype vector, and X_j_ denote the residual genotype matrix for the jth PLS component. w_j_ represents the first left singular vector of crossproduct matrix S_j_  =  X_j_^T^Y_j_ . u_j_ denotes the scores of X_j_ along the jth PLS component. During each iteration, the scores u_j_ was first calculated by
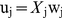
(2)


The loading a_j_ of Y_j_ and loading b_j_ of X_j_ at the jth PLS component were then defined by
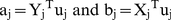
(3)


Finally, the residual phenotype vector Y_j+1_ and genotype matrix X_j+1_ for j+1 PLS component were calculated by
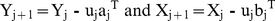
(4)


Suppose top m of ordered PLS components were further included into multilinear regression analysis. Let P_ij_ (j = 1,…,m) denote the score of subject i at the jth PLS component. PLS-based multilinear regression can be defined as
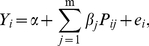
(5)


Where α is intercept. β_j_ denotes the regression coefficient for the jth PLS component. e_i_ denotes the residual environmental effect for subject i, and is assumed to follow a zero mean normal distributation with variance 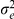. We can detect associations between target traits and candidate genetic regions under multilinear regression. For statistical tests, a permutation procedure is implemented here to obtain the empirical distribution of testing statistic of PLS-based MLAS in each replicate. The significance level of testing statistic of PLS-based MLAS can be evaluated according to the obtained empirical distribution.

### TSM-based MLAS

We also investigated the performance of TSM for MLAS. Suppose k genotyped SNPs within a candidate genetic region. Association tests can be conducted at each SNP, and p_(1)_≤p_(2)_ ≤…≤p_(k)_ denote the ordered p values of the k SNPs. TSM can be defined as
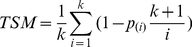
(6)


If none of SNPs within the candidate genetic region is associated with target traits, p_(i)_ should follow a beta distribution with expected value i/(k+1), and TSM should have an expected value 0. Otherwise, p_(i)_ will deviate from its expected value i/(k+1), and result in a positive value of 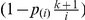. Large positive TSM value support the association between the candidate genetic region and target traits [24].

Taylor and Tibshirani showed that TSM approached normal distribution under large k (Equation 6), which could be used to determine the significance level of TSM statistic [24]. However, in a typical MLAS, the number of hypothesis test k (Equation 6) within a candidate genetic region is usually not large enough to approach normal distribution for TSM. In this study, we implemented a permutation procedure to estimate the empirical p value of TSM statistic.

### Simulations

Simulation study was used to assess the performance of our PLS-based MLAS as well as tagSNPs-based MLAS, PCA-based MLAS and TSM-based MLAS. HAPGEN program was used for genotype simulations [37], [38]. Based on known haplotype data, HAPGEN can simulate whole genome genotype data by implementing a hidden Markov model. Specific for this study, the phased haplotype data, minor allele frequencies (MAF) and D' of chromosome 6 of Caucasian were downloaded from the HapMap website (http://hapmap.ncbi.nlm.nih.gov/downloads/index.html.en). There were total 262,658 SNPs at chromosome 6. To simulate genes with various genetic structures, we randomly selected 10,000 genetic regions from chromosome 6. Each region contained 14 consecutive SNPs with 0.2≤MAF≤0.5 and pre-assigned ranges of D' between adjacent SNPs (Table 2). During each replicate, one of the 10,000 genetic regions was first randomly selected. HAPGEN was then used to simulate the genotype data of the selected genetic region with default running parameters recommended by HAPGEN developers [37], [38].

**Table 2:** Parameter configurations used in our simulation study.

		Genetic effect^a^		
Epistatic model	D'	SNP6	SNP10	SNP6×SNP10
	0.9∼1.0	0.020	0.010	0.000
	0.8∼0.9	0.018	0.008	0.004
	0.7∼0.8	0.016	0.006	0.008
	0.6∼0.7	0.014	0.004	0.012

		Genetic effect^b^
Additive model	D'	SNP8
	0.9∼1.0	0.01
	0.8∼0.9	0.02
	0.7∼0.8	0.03
	0.6∼0.7	0.04

a denote the phenotypic variance explained by additive effects of causal SNP 6 and SNP 10 as well as interactive effect between SNP 6 and SNP 10, respectively.

b denote the phenotypic variance explained by additive effect of causal SNP 8.

c the basic parameter configuration is highlighted in bold. Each possible parameter setting can be obtained by replacing one entry of the basic parameter configuration with a different entry of corresponding parameter.

Genetic epistatic and additive models were used for quantitative phenotype simulations. Let Y_i_ denote the trait value of subject i, defined by
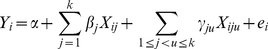
(7)where α is intercept. β_j_ denotes the additive effect of SNP j. X_ij_ (X_ij_  = 0,1 or 2) denotes the copy number of high risk allele for subject i at SNP j. Without loss of generality, we supposed that there was an interactive effect between the high risk alleles of SNP j and SNP u under the epistatic model.γ_ju_ denotes the interactive effect between SNP j and SNP u, and equate 0 in the additive model. For the epistatic model, X_iju_ was assigned 1 if the genotype vector of SNP j and SNP u was either of (2,2), (2,1) or (1,2), and 0 otherwise_._ e_i_ denotes the residual environmental effect of subject i, and follow a zero-mean normal distribution with variance 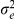. Under the epistatic model, SNP 6 and SNP 10 of the 14 SNPs were simulated as causal loci with additive effects and an interactive effect between SNP 6 and SNP 10. Under the additive model, SNP 8 of the 14 SNPs within selected genetic region was simulated as causal locus with additive genetic effect. Phenotypic variances and D' of the simulated genes were controlled to simulate various scenarios of association studies. Detailed parameter designs are presented in Table 2.

The simulated genotype (excluding causal SNP 6 and SNP 10 in the epistatic model and causal SNP 8 in the additive model) and phenotype data were simultaneously analyzed by tagSNPs-based MLAS, PLS-based MLAS, PCA-based MLAS and TSM-based MLAS. The F test was used here to compare the performance of various MLAS approaches. For tagSNPs-based MLAS, 3 of 12 SNPs (in the epistatic model) or 13 SNPs (in the additive model) were first selected as tagSNPs using hmmlsselect program [39]. The selected 3 tagSNPs were then included into the extended Tukey's 1-df interaction model (Equation 1) for MLAS using F test. For PLS-based MLAS and PCA-based MLAS, the first PLS and PCA components with the largest genotype variance in PLS and PCA analyses were included into multilinear regression analyses for MLAS in this study. For TSM-based MLAS, F test was first conducted at each SNP to obtain SLAS P values. The TSM statistic based on F test (FTSM) was then calculated for each gene (Equation 6). Additionally, the TSM statistic based on Wald test (WTSM) (implemented by PLINK [40]) was also calculated, and compared with FTSM to investigate potential impact of statistical tests on the performance of TSM-based MLAS. 2,000 permutations were conducted in each replicate to estimate the empirical p values of testing statistics of PLS-based MLAS, PCA-based MLAS and TSM-based MLAS.

5,000 replicates were conducted for each parameter setting. In each replicate, 800 individuals were simulated. Power and type I error rates were calculated respectively as the proportions of positive association results (P values ≤ 0.05) obtained from the simulated genes with and without genetic effects in 5,000 replicates. All our data simulations and analyses were implemented with statistical package R [35] except for WTSM (implemented by PLINK [40]).

### Application to Lean Body Mass GWAS Data

To investigate the efficiency of PLS-based MLAS, we applied it to a real GWAS data consisting of 1,000 unrelated US whites. The characteristics of this sample have been detailed in previous single locus GWAS of lean body mass [27]. Affymetrix 500 k SNP arrays were used to genotype a total of 500,568 SNPs. After quality control, 973 subjects and 379,319 SNPs relating to 12,828 genes were retained for our genome-wide MLAS of lean body mass. PLS-based MLAS approach was used to detect associations between each gene and lean body mass. 100,000 permutations were conducted to evaluate the empirical p value of each gene. To correct for multiple testing, false discovery rate (FDR) q value was also calculated from 100,000 permutations [41], [42]. Briefly, let 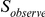 denote the observed PLS statistic vector with element 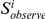 (i = 1,2,3…,12,828) in the lean body mass data. 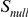 denotes the PLS statistic matrix with element 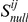 (i = 1,2,3…,12,828 and j = 1,2,3…,100,000), derived from 100,000 permutations. The FDR q value of gene m (denoted as 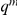, m = 1,2,3…,12,828) was calculated by
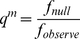
(8)where 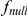 denotes the proportion of 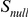 with 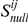
≥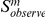, and 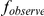 denotes the proportion of 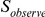 with 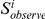
≥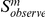 [41], [42]. Significant associations were defined by FDR q values≤0.05. Additionally, the FDR q value of each gene in previous single locus GWAS of lean body mass was also calculated with the qvalue package of R [35], [43], [44].
